# CT-guided percutaneous microwave ablation of pulmonary malignancies: Results in 69 cases

**DOI:** 10.1186/1477-7819-10-80

**Published:** 2012-05-07

**Authors:** Qiang Lu, Wei Cao, Lijun Huang, Yi Wan, Tonggang Liu, Qingshu Cheng, Yong Han, Xiaofei Li

**Affiliations:** 1 Department of Thoracic Surgery, Tangdu Hospital, The Fourth Military Medical University, Xi’an, People’s Republic China; 2 Department of Interventional Radiology, Tangdu Hospital, The Fourth Military Medical University, Xi’an, People’s Republic China; 3 Department of Health Statistics & Institute for Health Informatics, The Fourth Military Medical University, Xi’an, China; 4 Department of Respiratory, Tangdu Hospital, The Fourth Military Medical University, Xi’an, People’s Republic China

**Keywords:** Percutaneous microwave coagulation therapy, Inoperable pulmonary malignancies, Survival rates

## Abstract

****Background**:**

Microwave ablation (MWA) has attracted a worldwide attention gradually in treating inoperable pulmonary malignancies. However, in the lung tissues treated with MWA recurrence of tumor may still occur and few data in large patient groups till now were reported about the safety or effectiveness of microwave ablation in treating primary lung cancer and metastatic pulmonary malignancies. The purpose of this study is to evaluate the clinical curative effect (local control, survival data) MWA and its safety as well.

****Methods**:**

From 1 January 2005 to 1 January 2008, retrospective analyses, 69 patients underwent computed tomography (CT)-guided percutaneous MWA of pulmonary malignancies. All patients were deemed medically inoperable. The correlation of tumor sizes and local progression after ablation was analyzed and the survival rates within 3 years post surgery were compared between non-small-cell lung cancer and pulmonary metastases groups also.

****Results**:**

Pneumothorax was the most frequent complication and occurred in 24.64% patients after ablation. Neither needle track implantation was found nor did patient death occur in these patients within 30 days. The 1-, 2-, and 3-year overall survival rates were 66.7%, 44.9% and 24.6%, respectively. The overall survival rates for NSCLC patients in 1 year, 2 years, and 3 years were 75.0%, 54.2%, and 29.2%, respectively. The overall survival rates for pulmonary metastatic tumor patients in 1 year, 2 years, and 3 years were 47.6%, 23.8%, and 14.3%, respectively. The recurrence-free survival rates for NSCLC patients in 1 year, 2 years, and 3 years were 72.9%, 50.0%, and 27.1%, respectively. The mortality rates for pulmonary metastatic tumor patients in 1 year, 2 years, and 3 years were 47.6%, 19.0%, and 14.3%, respectively.

****Conclusions**:**

Percutaneous microwave coagulation therapy was one safe and effective method and could be beneficial for the improvement of inoperable pulmonary malignancies treatment effect.

## **Background**

Surgical operation is the preferred standard treatment for primary lung cancer patients [1,2]. However many patients were poor surgical candidates owing to insufficient cardiopulmonary function, old age, or other medical co-morbidities as well as those with pulmonary metastasis tumors, a life-threatening event with bad survival rates [3]. In recent years, tumor heating ablation such as radiofrequency ablation under the guidance of image has been proved to be an alternative treatment method for these patients with definite effects [4-8], and as a new minimally invasive technique, percutaneous microwave coagulation therapy (PMCT) has attracted a worldwide attention gradually in treating inoperable pulmonary malignancies.

Electromagnetic waves were used in microwave ablation (MWA) to produce tissue heating effects and would result in a much larger zone of active heating when compared with that of radiofrequency ablation, which made PMCT a more precise and more reliable method in treating malignancies in many tissues [9-11]. However few data in large patients groups till now were reported about the safety or effectiveness of MWA in treating primary lung cancer and metastatic pulmonary malignancies. The purpose of this study is to evaluate the clinical curative effect of MWA and its safety as well.

## **Methods**

### **Study design and setting**

This retrospective study was conducted in a single institution. Malignancies in all patients which included primary or recurrent non-small-cell lung cancer (NSCLC) and metastatic pulmonary malignancies were deemed medically inoperable. All patients underwent computed tomography (CT)-guided PMCT of pulmonary malignancies. The first PMCT procedures started in 2005. The endpoints were local efficacy, complications, and overall and disease-free survival.

From 1 January 2005 to 1 January 2008, retrospective analyses, 69 patients (45 males and 24 females; mean age 65 years ± 15 years (standard deviation)) underwent percutaneous MWA. Ninety-three intrapulmonary nodules were treated with PMCT (there were 106 intrapulmonary nodules detected by the CT, with 1.54 nodules for one patient on the average). With the agreement of Hospital Ethics Committee, all patients signed written informed consent before the treatment.

Inclusion criteria for patients receiving MWA of pulmonary malignancies were as follows: (1) age: 18–80 years; (2) having lost the opportunity of surgical resection or cannot endure surgical treatment because of other diseases (severe dysfunctions of heart, lung, liver, kidney, and so on); (3) with stage IIIB (according to UICC international NSCLC staging criteria) NSCLC or pulmonary metastasis tumor, and definite pathological diagnosis; or (4) patient refusing to undergo surgery.

Exclusion criteria were as follows: (1) patients with serious failure of the function of important organs (heart, liver, lung, and kidney); (2) patients with hilum pulmonis lesions and companied by larger cavity; (3) patients with central-type pulmonary malignancies and companied by severe obstructive pneumonia, patients with cancer involving main bronchus; (4) patients with pulmonary malignancies transferred to neck and thoracic vertebra, causing severe destruction of vertebral body and risk of paraplegia; or (5) patients with pulmonary diffuse metastatic lesions.

### **Image-guided microwave thermal ablation**

Ablation in all patients was performed with CT fluoroscopic guidance, with 5-mm collimation and 10–50 mA, to localize the tumor. Interventional radiologists performed lung MWA under the guidance of CT scan. Standard surgical prepping and draping was performed. Local anesthesia included 2% lidocaine both intradermally and deeper to the pleural surface. Patients were under conscious sedation using 0.5-1.0 mg doses of midazolam and 25–50 μg doses of fentanyl. Patients were monitored with continuous pulse oximetry and ECG. Blood pressure was measured every 5 min.

Percutaneous entry route was determined on the basis of tumor size, morphology, location, adjacent structures, and access route. Tumors that were <2 cm in maximal diameter were treated with a single applicator which was placed in the center of the tumor. To make the ablation range completely cover the tumors and 1 cm around them, tumors that were 2 cm or larger were treated with two to three different sites based on tumor size and shape. After the treatment, CT scan was performed again to define the change conditions of tumor and images of 1 cm range around it.

The microwave generator (Viva-Wave Microwave Coagulation System; Valley Lab) was capable of producing up to 60 W of power at a frequency of 915 MHz. A peristaltic pump perfused the outer shaft of the antenna with room-temperature normal saline at a rate of 60 mL/min to prevent thermal injury along the proximal antenna shaft. Ablation times were recorded for all procedures. Procedure success was determined by measuring an intratumoral temperature greater than 60°C or a sustained temperature of 55°C over more than one measurement. The total ablation time were adhered to in all cases, on the condition that the patients were able to tolerate.

Patients were observed for a minimum of 3 h after the procedure, with chest radiography performed at 2 h to assess for pneumothorax. Penicillin was administered prophylactically 3 days after MWA.

### **Complications and follow-up**

Treatment-related complications being counted were only within 30 days after ablation, and were classified in accordance with the Common Terminology Criteria for Adverse Events version 4.0 (CTCAE) [12]. Minor complications were defined as those resulting in no sequelae or needing nominal treatment or a short hospital stay for observation, which were Grade 1 or 2 adverse events. Major complications were defined as those resulting in readmission to the hospital for treatment, an unplanned increase in the level of care, extended hospitalization, permanent adverse sequelae, which were Grade 3 or 4 adverse events. Any patient death within 30 days of image-guided tumor ablation (grade 5 adverse events) was addressed.

Routine CT scan was performed after 1 month, then every 3 months for 3 years. Contiguously reconstructed sections (1:1 pitch) were obtained through the thorax in a single breath-hold, with 5-mm collimation. Median follow-up was 18 months. All CT scans during follow-up were done on the same CT unit and evaluated by the same group of radiologists. A modified Response Evaluation Criteria in Solid Tumors (RECIST) criterion incorporating CT scan was utilized to evaluate initial response to treatment [13]. Local progression was defined as any increase in the size of the measurements found on follow-up CT examinations. Stability or any decrease in size was considered as no local progression. Based on RECIST, quantitative analysis for local development of tumor was divided into: complete response (CR), partial response (PR), stable disease (SD), and progressive disease (PD) [13-15].

### **Statistical analysis**

Comparison of continuous data was performed by *t* test. Statistical analysis was performed by Chi-square test. Overall survival was calculated as the time between and the date of death or last news of the patient with the Kaplan-Meier technique and SPSS software, version 16.0 (SPSS, Chicago, IL, USA). Median follow-up time was calculated by the inversed Kaplan-Meier technique. Statistical significance was set at a *P* level of < 0.05.

## **Results**

### **MWA**

There were 26 patients of primary non-small-cell carcinoma pulmonary malignancies, 21 patients of metastatic pulmonary malignancies, and 22 patients of recurrent pulmonary malignancies in total. There were 47 patients with the diameter of 72 tumor nodules which were treated with PMCT less than 3 cm, 12 patients with the diameter of 12 tumor nodules more than 4 cm, and the diameter of the rest 19 tumor nodules was 3–4 cm, 12 patients in total. The average diameter of tumor nodules was about 22.3 ± 1.7 mm (8–55 mm). All patients received 3 years postoperative follow-up. For patient characteristics, see Table 1 (NSCLC) and Table 2 (pulmonary metastasis tumor).

**Table 1:** **Patient characteristics (NSCLC,*n*** **= 48)**

**Variable**	**Result**
Age, years, mean	67 ± 13
Sex, *n* (%)	
Male	33 (68.75)
Female	15 (31.25)
Pulmonary masses	56
NSCLC, *n* (%)	
Stage^a^ I	7 (14.58)
Stage^a^ II	10 (20.83)
Stage^a^ III	22 (45.84)
Stage^a^ IV	9 (18.75)
Previous therapy, *n* (%)	
No surgery^a^	26 (54.17)
Lung resection (recurrent)	22 (45.83)
Reasons for no surgery, *n* (%)	
Poor lung function	22 (45.83)
Sever cardiac risk	11 (22.92)
Poor performance status	8 (16.67)
Multiple co-morbidities	7 (14.58)
Length of hospital stay (days)	
Mean (range)	4.5 (3–27)

^a^Patients included without therapy, chemotherapy. or radiotherapy.

*NSCLC*, non-small-cell carcinoma.

**Table 2:** **Patient characteristics (pulmonary metastasis tumor,*n*** **= 21)**

**Variable**	**Result**
Age, years, mean	62 ± 12
Sex, *n* (%)	
Male	12 (57.14)
Female	9 (42.86)
pulmonary masses	37
Primary tumor, *n* (%)	
Breast cancer	3 (14.28)
Prostate cancer	4 (19.05)
Liver cancer	7 (33.33)
Gastrointestinal cancer	7 (33.33)
Previous therapy, *n* (%)	
No surgery^a^	11 (52.38)
Surgery	10 (47.62)
Reasons for no surgery, *n* (%)	
Poor lung function	12 (57.14)
Sever cardiac risk	5 (23.81)
Poor performance status	2 (9.52)
Multiple co-morbidities	2 (9.52)
Length of hospital stay (days)	
Mean (range)	4.8 (3–32)

^a^Patients included without therapy, chemotherapy, or radiotherapy.

### **Complications**

No needle track implantation and no patient deaths were found within 30 days after the operation. There were 17 out of 69 (24.64%) patients with complications following this procedure. Pneumothorax was the most frequent complication and occurred in 13 (18.84%) patients, including mild pneumothorax with no requirement for treatment (*n* = 8, 11.59%), thoracentesis (*n* = 4, 5.80%), and one (1.45%) severe pneumothorax received thoracic cavity closed drainage. Of these 13 patients who suffered pneumothorax, 10 patients had emphysema before ablation and two patients were accompanied by a small number of bullae. Other complications included five (7.25%) hemoptysis, two (2.90%) hemothorax, three (4.35%) pneumonitis, two (2.90%) chest pain without treatment, and two (2.90%) fever with temperature lower than 38.5°C (Table 3).

**Table 3:** Complications in 69 ablation patients

**Complication**	**Rate (%)**^**a**^
Pneumothorax	13 (18.84)
Mild	8 (11.59)
Moderate to severe^b^	5 (7.25)
Hemoptysis	5 (7.25)
Hemothorax	2 (2.90)
Pneumonia	3 (4.35)
Pain	2 (2.90)
Fever	2 (2.90)
Postablation syndrome	0
Acute respiratory distress syndrome	0

^a^The rate was calculated by dividing the number of ablation sessions in parentheses by the total number of ablation sessions (*n* = 69).

^b^Chest tube required.

### **Tumor progression**

Fifteen patients (21.74%) appeared with *in situ* local progress of masses. Among the 69 patients treated by MWA, the diameters of 47 patients were <3 cm, that of 12 patients were 3–4 cm, and that of the remaining 10 were >4 cm. During the MWA treatment for 91 pulmonary malignancies nodules, 178 different parts received MWA treatment in total. There were 2 ± 1 parts ablated for every tumor on the average.

Local progression rate of tumor within 3 years reached 16.13% (*n* = 15). There was no significant difference for local progression between tumors with diameters <3 cm and 3–4 cm (*P* = 0.373), but there was statistic difference between them and tumors with diameters >4 cm (*P* = 0.0396, *P* = 0.029, respectively) (Table 4).

**Table 4:** The relationship between sizes of tumor and local progression

**Tumor diameter**	**Patient number**	**Local control(CR, PR, SD),*n*(%)**	**Local progression(PD),*n*(%)**
<3 cm	47	38 (87.33)	9 (12.67)
3-4 cm	12	11 (91.67)	1 (8.33)
>4 cm	10	5 (50.00)	5 (50.00)

<3 cm *vs.* 3–4 cm, *x*^2^ = 0.794, *P* = 0.373; < 3 cm *vs.* >4 cm, *x*^2^ = 4.236, *P* = 0.0396; 3–4 cm *vs.* >4 cm, *x*^2^ = 4.774, *P* = 0.029; *CR*, complete response; *PD*, progressive disease; *PR*, partial response; *SD*, stable disease.

### **Survival**

**Figure 1:**
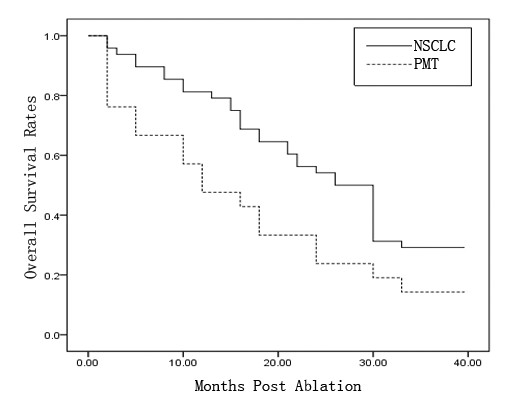
**Kaplan–Meier curve of overall survival rates for NSCLC and pulmonary metastasis tumor group treated with MWA. (*P*** **= 0.024).**

**Figure 2:**
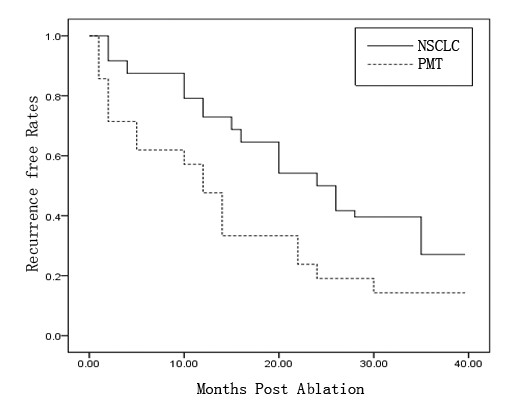
**Kaplan–Meier curve of recurrence free survival rates for NSCLC and pulmonary metastasis tumor group treated with MWA. (*P*** **= 0.015).**

The Kaplan-Meier median progress-free period for all patients (*n* = 69) was 22.0 months (95% confidence interval (CI) 15.023–28.977 months). The overall survival rates in 1 year, 2 years, and 3 years are 66.7%, 44.9%, and 24.6%, respectively; the overall survival rates for NSCLC patients in 1 year, 2 years, and 3 years were 75.0%, 54.2%, and 29.2%, respectively; and the overall survival rates for pulmonary metastatic tumor patients in 1 year, 2 years, and 3 years were 47.6%, 23.8%, and 14.3%, respectively (Figure 1). The median death period for all patients recurrence-free was 24.0 months (95% CI 17.828–30.172 months). The recurrence-free survival rates for NSCLC patients in 1 year, 2 years, and 3 years are 72.9%, 50.0%, and 27.1%, respectively; and the mortality rates for pulmonary metastatic tumor patients in 1 year, 2 years, and 3 years are 47.6%, 19.0%, and 14.3%, respectively (Figure 2). There were no significant differences in either the overall survival rates or the recurrence-free survival rates for NSCLC and pulmonary metastatic tumor patients.

## **Discussion**

The preferred therapeutic schedule for pulmonary malignancies is surgical treatment, but for those inoperable pulmonary malignancies patients who cannot endure surgical treatment because of various reasons (such as advanced pulmonary malignancies, recurrent pulmonary malignancies, metastatic pulmonary malignancies, and so on), various comprehensive treatment methods had to be carried out to improve the long-term survival rates of such patients [16-18]. Tumor heating ablation under the guidance of image has been proved to be one treatment method with definite effects [19,20], and the method of treating inoperable pulmonary malignancies with percutaneous microwave coagulation therapy (PMCT) is one of the new minimally invasive technique popular in recent years [21,22].

MWA uses electromagnetic waves to produce tissue heating effects and would result in a much larger zone of active heating when compared with that of radiofrequency ablation for its greater convection profile in lung and less severe heat sink effects. Furukawa *et al.* also found that tissues around the electrodes changed immediately after MWA, that is fibrosis and thickening of collagenous fibre [23]. The ablated pulmonary tissues would be replaced by scar fibrous tissues after 6 months.

The clinical safety of MWA has been proved in some clinical studies. Wolf [24] reported that there was no patient death within 30 days after the operation, and the first death appeared 256 days after MWA because of a fatal massive hemoptysis. He *et al.*[25] treated 16 tumors in 12 patients with MWA therapy under the guidance of ultrasound, and follow-up was carried out for 20 months on the average. In this group, seven patients survived with no severe complications, and five patients died of metastasis. Nevertheless more data, especially in large patient group, were still needed for evaluating the safety and effectiveness of MWA in treating primary pulmonary malignancies and metastatic pulmonary malignancies.

In our group, all patients survived within 30 days, and complications occurred in 17 patients (24.64%). The most common complication after percutaneous PMCT treatment was pneumothorax caused by pulmonary needle biopsy, and the incidence rate of pneumothorax was about 15% to 45% [22,24,26,27]. In our study, the incidence rate of pneumothorax was 18.84%. Of these, 13 patients suffered pneumothorax, 10 patients had emphysema before ablation, and two patients were accompanied by a small number of bullae, so it was suggested that emphysema and bullae may be the risk factors of pneumothorax in PMCT treatment. However for those patients, who had a small amount of pneumothorax, no treatment was needed and the incidence rate of thoracic cavity puncture and closed drainage was 5.80% and 1.45%, respectively. Other common complications after PMCT operation include blood-stained sputum, hemothorax and pulmonitis, and so on. The incidence rate of chest pain within 1–2 h after microwave ablation was 2.90%, all of which were patients with tumors close to the thoracic wall.

The first patient died of massive hemoptysis 207 days after the MWA therapy in our group. The pulmonary malignancies in this patient were located in the periphery of the pulmonary artery, with a diameter of 4 cm, and the patient received 3x MWA on different parts during the operation. A larger cavity was found in the CT scan 3 month after the operation, which may be the primary cause of massive hemoptysis. It was found that the tumor position in almost all patients that appeared as blood-stained sputum after operation was adjacent to bronchia, pulmonary artery, and pulmonary vein, which suggested that it was a risk factor for massive hemoptysis when the tumor was located near the bronchia, pulmonary artery, and pulmonary vein. Therefore appropriate criteria should be set for patients receiving PMCT treatment, and a well-trained and experienced clinician was needed to reduce the complications when performing PMCT.

The clinical efficacy of microwave ablating was the most valuable fact in treating inoperable pulmonary malignancies. In early reports, the local progress rates of pulmonary malignancies after PMCT were confused [24,28,29]. The median progress-free period, survival rate in 1 and 2 years and tumor’s local progress in our study suggested that MWA therapy was definitely effective, especially for patients with non-metastatic pulmonary malignancies. For patients with metastatic pulmonary malignancies, the result was unsatisfied, which might be owing to later cancer staging and poor general conditions.

In these 69 patients in our study, local progresses occurred in 15 cases (21.74%), which showed that local progresses of pulmonary malignancies after PMCT treatment was still a mean problem for PMCT. In further study, it was found that a possible reason might be relevant to the tumor size; the larger the tumor was, the higher the recurrent rate was. Wolf *et al.*[24] reported that for patients with tumor diameter >3 cm, 26% of them had a residual tumor at MWA parts, 22% of them had recurrent tumor. In our group, among 69 cases of ablated masses, all the five cases whose masses were >4 cm in diameter before treatment had local progress after the PMCT, and patients with mass greater than 4 cm in diameter had a significantly higher ratio of local progress compared with masses of other sizes, which suggested that when the tumor mass was >4 cm in diameter it seemed to be a risk factor for the local tumor progress and might be exclusion criteria for microwave treatment.

## **Conclusion**

Ablating pulmonary malignancies with microwave was a safe and effective method, which could obviously be beneficial for the improvement of pulmonary malignancies treatment effect although further observation was still needed for its long-term effect.

## **Abbreviations**

CR: Complete response; CT: Computerized tomography; CTCAE: Common Terminology Criteria for Adverse Events; ECG: Electrocardiogram; MWA: Microwave ablation; NSCLC: Non-small-cell lung cancer; PD: and progressive disease; PMCT: Percutaneous microwave coagulation therapy; PMT: Pulmonary metastasis tumor; PR: Partial response; RECIST: Response Evaluation Criteria in Solid Tumors; SD: Stable disease; SPSS: Statistical Product and Service Solutions.

## **Competing interests**

All of the authors in this manuscript declared that no financial or non-financial interests were related to this study or future application after publication.

## **Authors’ contributions**

QL was responsible for manuscript writing (First Author); WC and LH supervised QL to write this manuscript and gave comments to revise it (co-author); YH and XL were responsible for whole project design (Corresponding Author); YW is in charge of data analysis; TL and QC is in charge of correction of manuscript. All authors read and approved the final manuscript.
